# Optimal design for a hybrid microgrid-hydrogen storage facility in Saudi Arabia

**DOI:** 10.1186/s13705-022-00351-7

**Published:** 2022-05-27

**Authors:** Abdulaziz A. Alturki

**Affiliations:** Department of Chemical and Materials Engineering, Faculty of Engineering-Rabigh Branch, King Abdulaziz University, Jeddah, 21589 Saudi Arabia

**Keywords:** CO_2_ emissions, Hybrid microgrid, Carbon management, Green hydrogen, Renewable energy

## Abstract

**Background:**

Sustainable development requires access to affordable, reliable, and efficient energy to lift billions of people out of poverty and improve their standard of living. The development of new and renewable forms of energy that emit less CO_2_ may not materialize quickly enough or at a price point that allows people to attain the standard of living they desire and deserve. As a result, a parallel path to sustainability must be developed that uses both renewable and clean carbon-based methods. Hybrid microgrids are promoted to solve various electrical and energy-related issues that incorporate renewable energy sources such as photovoltaics, wind, diesel generation, or a combination of these sources. Utilizing microgrids in electric power generation has several benefits including clean energy, increased grid stability, and reduced congestion. Despite these advantages, microgrids are not frequently deployed because of economic concerns. To address these financial concerns, it is necessary to explore the ideal configuration of microgrids based on the quantity, quality, and availability of sustainable energy sources used to install the microgrid and the optimal design of microgrid components. These considerations are reflected in net present value and levelized energy cost.

**Methods:**

HOMER was used to simulate numerous system configurations and select the most feasible solution according to the net present value, levelizied cost of energy and hydrogen, operating cost, and renewable fraction. HOMER performed a repeated algorithm process to determine the most feasible system configuration and parameters with the least economic costs and highest benefits to achieve a practically feasible system configuration.

**Results:**

This article aimed to construct a cost-effective microgrid system for Saudi Arabia's Yanbu city using five configurations using excess energy to generate hydrogen. The obtained results indicate that the optimal configuration for the specified area is a hybrid photovoltaic/wind/battery/generator/fuel cell/hydrogen electrolyzer microgrid with a net present value and levelized energy cost of $10.6 billion and $0.15/kWh.

**Conclusion:**

With solar photovoltaic and wind generation costs declining, building electrolyzers in locations with excellent renewable resource conditions, such as Saudi Arabia, could become a low-cost hydrogen supply option, even when accounting for the transmission and distribution costs of transporting hydrogen from renewable resource locations to end-users. The optimum configuration can generate up to 32,132 tons of hydrogen per year (tH_2_/year), and 380,824 tons per year of CO_2_ emissions can be avoided.

## Background

The world's energy demand is expanding at a breakneck pace and is expected to increase by up to 55% by 2035 [1]. Energy is critical in modern society, and conventional fossil fuels are rapidly depleting because of rapid industrialization and population growth. Notably, fossil fuels such as gasoline, natural gas, and coal used to generate electricity have contributed significantly to global climate change, affecting millions of people daily. Additionally, the 2015 Paris climate change summit emphasized the importance of all countries decreasing carbon emissions [2]. Fossil fuel depletion, environmental concerns, and the dangers associated with nuclear energy all compel the international community to embrace renewable energy (RE) sources, primarily the isolated mode in nonelectrified areas where grid extension is costly. Otherwise, the intermittency of renewable resources is managed.

The exponential rise in global energy demands and the utilization of conventional energy resources are the primary reasons for the extreme increase in global greenhouse gas (GHG) emissions. The most efficient way of solving this problem is to utilize RE resources such as solar photovoltaic (PV), wind, and hydro or combine conventional and RE resources. Using hybrid systems such as PV and wind energy is viewed as complementary. These hybrid energy systems fulfill energy demands with the least GHG emissions and contribute to the global cause of environmental protection. The utilization of solely RE resources for energy generation is a better option, but the high prices of renewable systems are making this option unsuitable for places such as the Middle East, where fuel prices are much lower than those in other areas, and using hybrid systems is viable in this area. H_2_ technology has acquired increased popularity in recent years as a result of the advancement of fuel cells (FCs) and is anticipated to have a bright future [3, 4]. The use of H_2_ to energize FCs indicates that H_2_ outperforms diesel generator systems, which are typically utilized for long-term storage. FCs constitute a low-maintenance, high-performance, emission-free technology [3]. H_2_ can be created via electrolysis and stored in H_2_ tanks; this is referred to as RE storage [5]. The H_2_ tank, as an energy storage system, captures extra energy via the electrolyzer and injects it into the FC to compensate for generation shortfalls. The addition of FC to hybrid RE systems reduces the BT size, increases the BT lifespan, and enhances the overall system performance [6]. Thus, regarding RE systems, whether stand-alone or grid-connected, the primary difficulty is scaling the system components and developing an effective energy management strategy (EMS) that meets the intended objectives without jeopardizing system performance [7, 8]. In other words, component size and EMS are the primary determinants of the RE system's operational and start-up expenses.

Hybrid microgrid systems (HMGs) have become critical for rural electrification. Numerous studies (e.g., [9–16]) have investigated and proposed a hybrid renewable energy system (HRES). These studies provide all the required information for designing isolated HRESs. The authors of [9] discuss the design and financing of a microgrid on the tiny island of Koh Jik. HOMER software application was utilized to gain insight into the techno-economic environment. Similarly, lead–acid and lithium-ion battery technologies were compared regarding their impact on the levelized cost of energy (LCOE) and renewable portions.

The authors of [10] provide an overview of the characteristics of energy system models and the available tools for optimizing multi-energy systems. The authors of [11] provide an overview of new optimization techniques for addressing operational costs and minimizing overall network losses. The authors of [12] provide an overview of system optimization and energy management strategies for PV, wind turbine, and fuel cell energy sources. Another study [13] describes the development of microgrids in seaport areas. Similarly, the authors of [14] provide an overview of the fundamental difficulties surrounding HRES adoption and a study of the various RE sources that can be combined in isolated and grid-connected modes. The authors of [15] present a study of HRES design optimization methods, limitations, and battery types. Finally, the authors of [16] created a cost-effective microgrid system for the Saudi Arabian city of Yanbu. Their design uses the optimal microgrid configuration while minimizing net present cost (NPC) and LCOE under certain technical conditions such as the probability of power supply failure and the availability index. The author of [17] proposes a novel application of the equilibrium optimizer (EO) for designing a hybrid microgrid to supply electricity to Dakhla, Morocco, a remote area. EO was chosen to design the microgrid system due to its high effectiveness at quickly determining the optimal solution. EO is utilized for the purpose of determining the optimal system design that minimizes cost, increases system stability, and covers the load under a variety of climate conditions. The results indicate that the proposed EO achieves the optimal system design, with RE sources (PV and wind turbine [WT]) accounting for 97% of the annual contribution and EO exhibiting fast convergence characteristics. The best NPC, LCOE, and LPSP are achieved through EO at $74,327, $0.0917/kWh, and 0.0489, respectively. The author of [18] used a microgrid to serve a load installed in a remote area of Saudi Arabia's Aljouf region using a social spider optimizer (SSO) to determine the optimal size of a HRES integrated microgrid (MG) consisting of PV solar panels, WT, a battery, a diesel generator (DG), and an inverter. With a cost of energy (COE) of $0.1349/kWh and a LPSP of 0.01714, the proposed SSO provides the optimal architecture for a HRES integrated microgrid.

This current study is conducted to investigate and select the best hybrid energy generation system in the region of the Kingdom of Saudi Arabia (KSA), where the main target is the least carbon emissions while maintaining economic viability and producing hydrogen. Different system configurations are considered in this study to find the optimal energy generation system with a total generation capacity of approximately 1 GW. KSA generates more electricity than any other country in the Middle East, with an estimated 362 terawatt hours in 2019, roughly the same as in 2018. From 2000 to 2015, power generation increased at an average annual rate of 6%, but growth slowed significantly as the population grew. Economic growth slowed from 2016 to 2018; energy efficiency and demand-side management measures were implemented, and electricity prices increased.

According to data from BP's Statistical Review of World Energy 2021, power generation fell by 1% in 2020 due to the economic slowdown brought about by the COVID-19 pandemic [19]. Due to pandemic-related lockdowns and restrictions, residential energy consumption increased, but commercial and government electricity sales decreased. KSA currently has a generation capacity of 79.67 GW [20], and electricity demand continues to grow at a rate of 3000 megawatts per year. In comparison to other countries, this annual growth rate is relatively high. Currently, the cost of producing electricity is $0.10/kWh, and the average price of electricity is approximately $0.03/kWh. The question here is whether the low prices are subsidized or simply low prices. According to the International Energy Agency (IEA), governments spend fuel subsidies to keep fuel prices lower than the cost of production for their citizens. Government funds cover the difference between prices and costs. These continuous high price subsidies impose an economic burden on the government. Thus, integrating RE resources with the current power generating system can be an excellent solution because they consume no fossil fuel, which eliminates the need for subsidies. As a result, a great deal of the costs could be saved as they would not be consumed as subsidies. RE power systems have high initial costs, but return this initial investment with free green energy generation for many years. KSA, in general, is an excellent location for solar energy. KSA receives approximately 250 w/m^2^ of solar radiation annually, nearly double the amount received in most of the world [21]. This also equates to 105 trillion kWh per day or roughly 10 billion barrels of crude oil in energy terms.

Solar energy is booming due to Saudi Vision 2030s economic diversification plans. KSA has committed to installing 27.3 GW of RE by 2023, the majority of which (20 GW) will be solar PV, while the remaining 7.3 GW will be wind and concentrated solar power (CSP). By 2030, solar PV, wind, and CSP will total 40 GW, 16 GW, and 2.7 GW, respectively. This target corresponds to approximately 32% of the current peak power consumption in KSA (estimated at 61.743 GW in 2018). This high level of solar generation is possible due to the significant decrease in the cost of solar PV technology [22]. In addition to the ambitious renewable plans, KSA is building its first green hydrogen plant, which is expected to cost SR19 billion ($5 billion). The plant will be located in Neom—the planned city being constructed in the Tabuk province of northwestern KSA—and powered by 4 GW of wind and solar energy; it is scheduled to begin production in 2025 [23].

Microgrid design and power management are examined in this article for five configurations—generator–PV–wind–battery, generator–wind–battery, generator–PV–battery, generator–battery, and generator only—to supply an isolated area in KSA’s Yanbu region. A load of around 1.2 GW is taken to generate constant 1 GW while compensating for power system and transmission losses. The grid was not integrated as we aimed to have a 100% RE system with the least carbon emissions, and we also wanted to generate hydrogen through pure RE. The load-following strategy was used in the paper as a control strategy. The primary objective aimed for in this paper is to minimize NPC while considering technical constraints such as the integration of solar PV, wind, or a combination of both into a conventional diesel power plant to create a generating system with the least carbon emissions. In this work, we aim to study the feasibility of using an integrated RE system and traditional power plants to find the optimal system configuration. The system configuration with the highest renewable penetration and minimum fuel consumption is selected as the best system. The overall performance of the optimal system configuration can be enhanced further by utilizing any excess energy generation that is not consumed by the load into hydrogen production, thus increasing the overall system efficiency and reliability. In summary, the article makes four significant contributions:

- Optimal design of a microgrid system feeding a load in Saudi Arabia's Yanbu region,
- Proposal and analysis of five microgrid configurations in terms of their technical and operational characteristics,
- Presentation of the hybrid renewable microgrid system's optimal design and operation through the selection of suitable renewable sources to meet the system's objectives and constraints.
- Produce hydrogen via the optimum microgrid design.

This paper is structured as follows: “Methods” discusses the modeling of the system (PV–Wind–Generator (Gen)–ESS, Wind–Gen–ESS, PV–Gen–ESS, Gen–ESS, and Gen only) and provides an overview of the optimization problem function (NPC, LPSP, and LCOE). “Results” describes the location and specifications of the system under investigation. “Discussion” presents a comparative analysis of the various optimization configurations. “Conclusion” concludes the paper.

## Methods

Several simulation techniques are present in the literature to study RE systems' characteristics including feasibility, simulation, techno-economic, and optimization analysis. Different software tools cover various aspects of RE systems and provide an essential ground to determine whether to utilize RE systems in power generation for a particular location. RET-Screen is the software used for prefeasibility studies; it computes and compares the economic aspects of the renewable system to the conventional approach [24]. It highlights the advantage of one method over another, but this software's deficiency is that it does not optimize. The HYBRID2 simulation utilizes statistical and time-series methodologies to evaluate HRES performance [25]. Despite being precise in simulation, this software also lacks the optimization performance capability. TRNSYS is another well-known transient simulation software tool for thermoelectrical systems. However, this software lacks a primary, user-friendly interface, so great difficulty occurs when working with this software [26]. It can be concluded that neither RET-Screen, HYBRID2, nor TRNSYS can be utilized for efficient techno-economic and optimization analysis of RE systems.

**Fig. 1:**
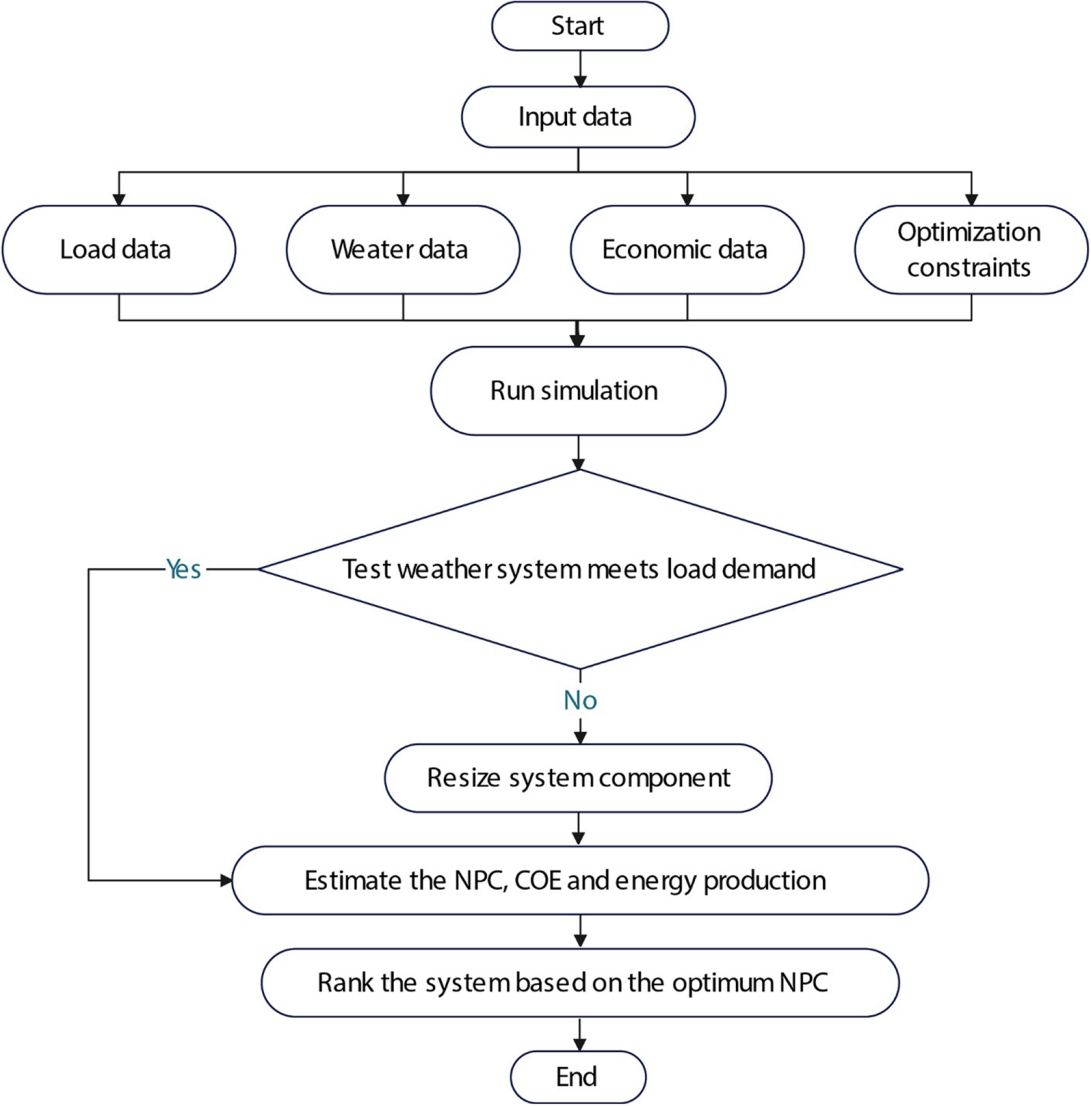
Optimization flowchart for HOMER

HOMER can perform all the required analyses with extreme precision and in the most user-friendly way [27]. This software can be efficiently utilized for performing techno-economic and environmental analyses on a wide range of power generation systems. It can also perform optimization to find an optimal solution for the system. It can simulate numerous system configurations and select the most feasible solution according to the chosen parameters. The parameters can include the NPC, COE, operating cost and renewable fraction (RF), GHG emissions, etc. It performs a repeated algorithm process to determine the most feasible system configuration and parameters with the least economic costs and highest benefits. It addresses technical aspects while optimizing to achieve a practically feasible system configuration. Figure 1 shows an optimization flowchart for HOMER.

Techno-economic analysis is conducted under the assumption that the prices of system components increase at the same rate. To carry out the calculation, the location-specific nominal interest rate (′*i*) and annual inflation rate (*f*) are taken into account. The project’s duration is estimated to be 25 years [28]. HOMER Pro calculated the net present value (NPV) as follows:

1 $$\mathrm{NPC}= \sum_{n=1}^{t}{i}_{\mathrm{d}}\left({C}_{\mathrm{Com}}+{C}_{\mathrm{Rep}}+{C}_{\mathrm{O}\&\mathrm{M}}+{C}_{\mathrm{Fuel}}+{C}_{\mathrm{Sal}}\right)$$

where ${i}_{\mathrm{d}}$ is the discount rate; $n$ is the project lifetime in years; ${C}_{\mathrm{Com}}$ is the capital cost of a system component $; {C}_{\mathrm{Rep}}$ is the replacement cost; ${C}_{\mathrm{O}\&\mathrm{M}}$ is the operation and maintenance cost; ${C}_{\mathrm{Fuel}}$ is the fuel cost; and ${C}_{\mathrm{Sal}}$ is the salvage value. The following formula can be used to determine the discount rate (${i}_{\mathrm{d}}$):

2 $${i}_{\mathrm{d}}=\frac{1}{{\left(1+i\right)}^{n}},$$

where $i$ is the real annual interest rate, which can be calculated as follows using the nominal interest rate (${i}^{^{\prime}}$) and annual inflation ($f$) [29]:

3 $$i=\frac{{i}^{^{\prime}}-f}{1+f}.$$

The annualized cost is calculated in HOMER by first calculating the net present value (NPV) of the project and then multiplying it by the capital recovery factor (CRF), as shown in the following equation:

4 $${C}_{\mathrm{ann}}=\mathrm{CRF}\left(i,n\right)\times \mathrm{NPC}.$$

The $\mathrm{CRF}$ is a ratio used to determine an annuity's present value (a series of equal annual cash flows). The $\mathrm{CRF}$ equation is as follows:

5 $$\mathrm{CRF}\left(i,n\right)=\frac{i{\left(1+i\right)}^{n}}{{\left(1+i\right)}^{n}-1}.$$

In HOMER, the LCOE is calculated as follows:

6 $$\mathrm{LCOE}=\frac{{C}_{\mathrm{ann},\mathrm{tot}}-{c}_{\mathrm{boiler}}{H}_{\mathrm{served}}}{{E}_{\mathrm{served}}},$$

where ${C}_{\mathrm{ann},\mathrm{tot}}$ represents the system's total annualized cost; ${c}_{\mathrm{boiler}}$ represents the boiler's marginal cost; ${H}_{\mathrm{served}}$ represents the total thermal load served; and ${E}_{\mathrm{served}}$ represents the total electrical load served.

In HOMER Pro, the following equation estimates the levelized cost of hydrogen (LCOH):

7 $$\mathrm{LCOH}= \frac{{C}_{\mathrm{ann},\mathrm{tot} }- {v}_{\mathrm{elec}}\left({E}_{\mathrm{prim},\mathrm{AC}}-{E}_{\mathrm{prim},\mathrm{DC}}-{E}_{\mathrm{def}}+{E}_{\mathrm{grid},\mathrm{sales}}\right)}{{M}_{{H}_{2}}},$$

where ${C}_{\mathrm{ann},\mathrm{tot}}$ is the total annualized cost, ${v}_{\mathrm{elec}}$ represents the value of electricity, ${E}_{\mathrm{prim}}$ represents the principal electrical load, ${E}_{\mathrm{def}}$ represents the deferrable load, ${E}_{\mathrm{grid}}$, sales represents the total energy sold to the grid, and ${M}_{{H}_{2}}$ represents the total hydrogen generation.

### Mathematical modeling

In this study, the most crucial determination for finalizing the optimal system configuration is finding the system with the least GHG emissions and the highest RF. The RF can be calculated using Eq. 1. RF can also be calculated by HOMER; it is the amount of energy produced from the RE resources compared to the total energy produced by the whole system [30]:

8 $$\mathrm{RF}=\frac{{E}_{\mathrm{ren}}+{T}_{\mathrm{ren}}}{{E}_{\mathrm{pro}}}.$$

Here, ${E}_{\mathrm{ren}}$ is the energy generated from RE resources, whereas ${T}_{\mathrm{ren}}$ represents the thermal energy generated from renewable resources. ${E}_{\mathrm{pro}}$ is the total energy generation of the system from either renewable or nonrenewable resources.

Techno-economic and environmental analyses of the various power systems are conducted to select optimal approaches. Different system configurations are compared to highlight the effectiveness of utilizing RE systems regarding the environment. This comparison also highlights the economic and technical aspects of different system configurations. This environmental analysis is based on identifying the configuration with the least GHG emissions. The techno-economic analysis helps finalize the most suitable configuration while following all the techno-economic and environmental constraints. Five configurations are studied:

- Generator–PV–wind–battery,
- Generator–wind–battery,
- Generator–PV–battery,
- Generator–battery,
- Generator only.

**Fig. 2:**
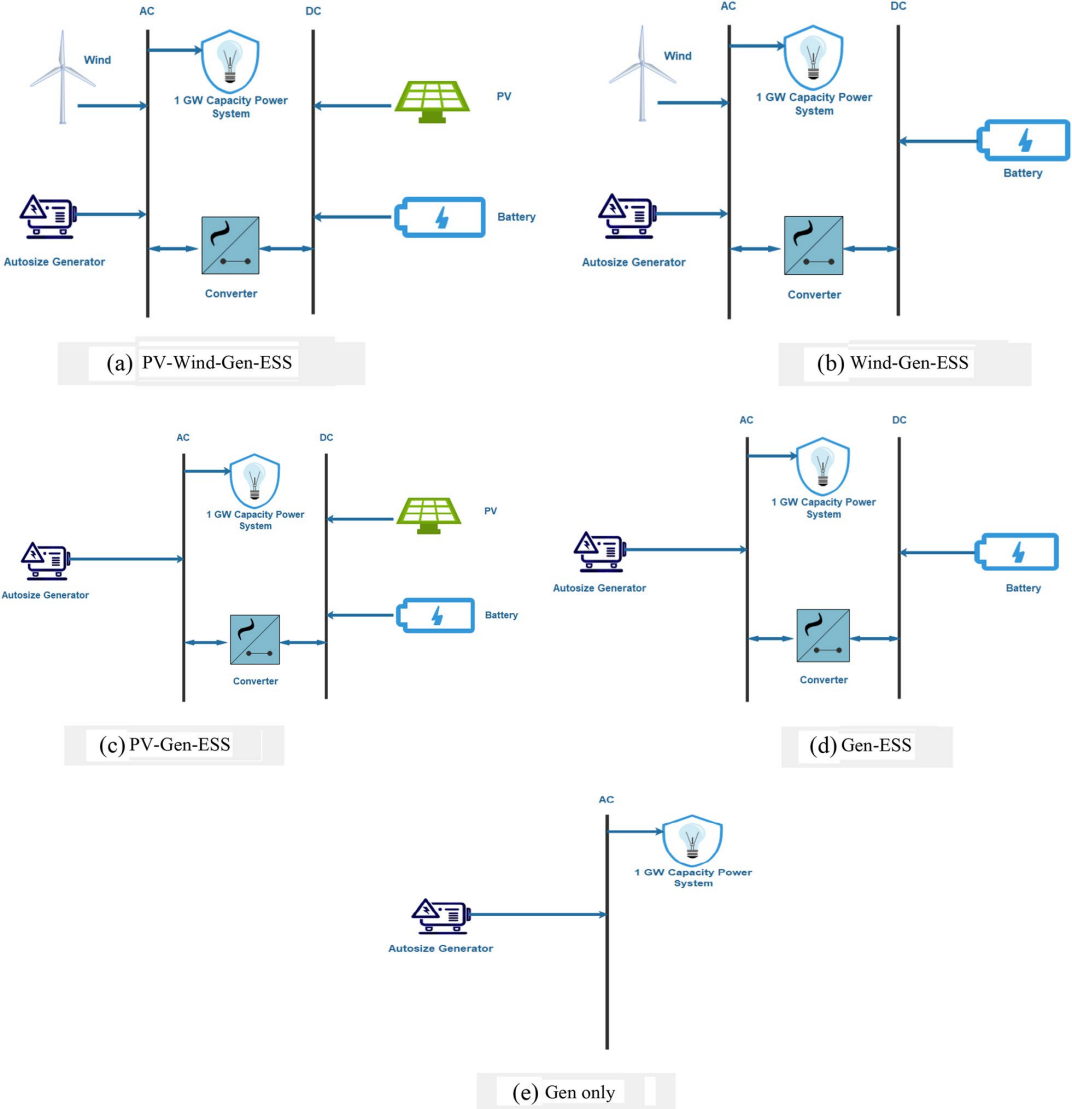
Schematic representation of different system configurations

These configurations were selected to find an optimal solution to generate the desired amount of energy with the least COE and NPC and the highest RF while producing the least GHG emissions. The battery is used as an energy-storing source (ESS). The considered configurations are presented in Fig. 2.

### Solar and wind potential at Yanbu, KSA

**Fig. 3:**
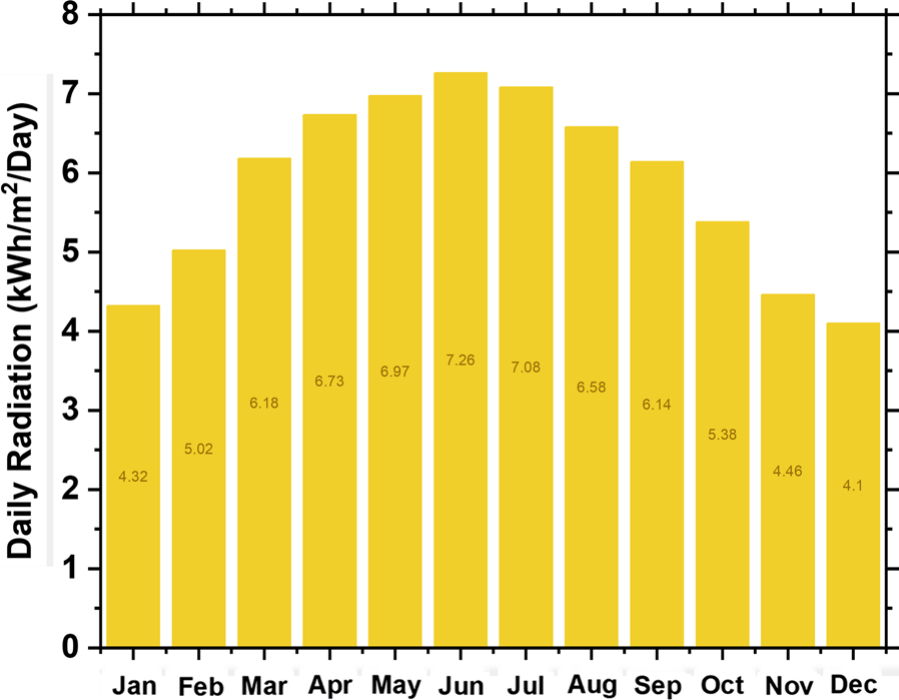
Seasonal solar potential

By connecting to the NASA Atmospheric Science Data Center, HOMER can extract the required weather data based on the longitude and latitude of a specific location. Monthly averages of global solar radiation and wind speed are shown in Figs. 2 and 3 for the locations mentioned previously. Typically, the nature of load demand has a significant impact on system reliability, and thus load data are synthesized by integrating randomness with daily and hourly noise inputs over the last two years of monthly electric load profiles. Typically, HOMER Pro accumulates a year's worth of load data (i.e., 8,760 hourly values) from specific daily profiles at a specific location.

The PV panel output can be expressed as follows [31]:

9 $${P}_{\mathrm{pv}}=I\left(t\right)\times {\eta }_{\mathrm{pv}}\times {A}_{\mathrm{pv}},$$

where $I$ denotes solar irradiation; ${A}_{\mathrm{pv}}$ denotes the area of the PV panel; and ${\eta }_{\mathrm{pv}}$ denotes the PV system's efficiency, which is calculated as follows:

10 $${\eta }_{\mathrm{pv}}={\eta }_{\mathrm{r}} \times {\eta }_{\mathrm{t}}\times \left[1-\beta \times \left({T}_{\mathrm{a}}\langle t \rangle -{T}_{\mathrm{r}}\right)-\beta \times 1<t>\times \left(\frac{\mathrm{NOCT }- 20}{800}\right)\times \left(1-{\eta }_{\mathrm{r}}\times {\eta }_{\mathrm{t}}\right)\right],$$

where NOCT denotes the nominal operating temperature of the cell (°C); ${\eta }_{\mathrm{r}}$ denotes the reference efficiency; ${\eta }_{\mathrm{t}}$ denotes the maximum power point (MPPT) equipment efficiency; $\beta$ is the temperature coefficient; ${T}_{\mathrm{a}}$ denotes the ambient temperature (°C); and ${T}_{\mathrm{r}}$ denotes the cell reference temperature (°C).

In Yanbu, KSA, the PV potential is approximately 5.85 kWh/m^2^/day. The highest solar irradiance is in June, while the lowest is in December. The system can generate the highest solar energy in June and the lowest in December. The solar potential of the site is presented in Fig. 3.

Since there are numerous manufacturers of energy system components on the market, we calculated an average component cost as an input using data from the literature and manufacturers [32–39]. The specifications of the solar system used in this study are presented in Table 1. A 100-kW solar system is considered to be a standard PV system that contributes to the total energy generation of 1 GW.

**Table 1:** Parameters of the solar PV system [32–39]

Rated power	100 kW
Capital cost	$90,000
Replacement cost	$90,000
O&M cost/year	$1,000
Lifetime	25 years

Wind power is proportional to wind speed, which can be expressed as follows [40]:

11 $${P}_{\mathrm{wind}}=\left\{a\times V \times {<t>}^{3}-b\times \begin{array}{c}0, V<t>\le {V}_{\mathrm{Ci}}, V<t>\ge {V}_{\mathrm{CO}}\\ {P}_{\mathrm{r}},{V}_{\mathrm{Ci}}< V<t>{<V}_{\mathrm{r}}\\ {P}_{\mathrm{r}},{V}_{\mathrm{r}}\le V<t>{<V}_{\mathrm{CO}}\end{array}\right\},$$

where $V$ denotes wind speed; ${P}_{\mathrm{r}}$ denotes wind-rated power; ${V}_{\mathrm{Ci}}$, ${V}_{\mathrm{CO}}$, and ${V}_{\mathrm{r}}$ denote cut-in, cut-out, and rated wind speeds, respectively; and $a$ and $b$ represent two constants calculated as follows:

12 $$\left\{\begin{array}{c}a= {P}_{\mathrm{r}}/({{V}_{\mathrm{r}}}^{3}-{{V}_{\mathrm{Ci}}}^{3})\\ b= {V}_{\mathrm{Ci}}/({{V}_{\mathrm{r}}}^{3}-{{V}_{\mathrm{Ci}}}^{3})\end{array}.\right.$$

The rated power of wind is calculated in the following manner:

13 $${P}_{\mathrm{r}}= \frac{1}{2} \times \rho {\times A}_{\mathrm{wind}}\times {C}_{\mathrm{p}}\times {V}_{\mathrm{r}}^{3},$$

where $\rho$ denotes the air density; ${A}_{\mathrm{wind}}$ denotes the area swept by a wind turbine; and ${C}_{\mathrm{p}}$ represents the maximum power coefficient, which must be from 0.25 to 0.45%.

**Fig. 4:**
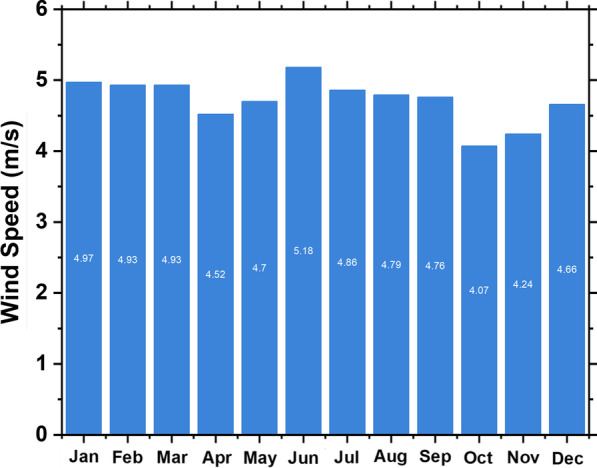
Seasonal wind speed (m/s)

The wind potential at Yanbu is sufficient to be considered for power generation. The annual average wind speed on a scaled basis is 4.72 m/s, which is sufficient for energy generation through wind potential. The highest wind speed occurs in July, whereas the lowest wind speed is in October. Wind potential is depicted in Fig. 4. The specifications of the wind turbine considered in this study are illustrated in Table 2.

**Table 2:** Wind turbine specifications [32–39]

Rated power	1.5 MW
Capital cost	$2,000,000
Replacement cost	$2,000,000
O&M cost/year	$20,000
Rated power	20 years

These RE potentials make it clear that a HRES can be installed at this location for power generation. The energy demand can also be fulfilled by utilizing conventional power plants solely, such as coal and fossil fuel-based power plants. However, this approach will adversely affect the environmental wellbeing of this area, as these power plants will create hazardous emissions. Therefore, a HRES approach is inevitable to solve the problem of the energy crisis so that with the power demand fulfillment, the environment is saved from emissions, and a green, sustainable way of generating energy is approached. The criteria mentioned above make this area a perfect candidate for a HRES project.

### Battery and charge controller

Battery storage is a critical component of isolated microgrid systems. The capacity of the battery (in kWh) can be expressed as follows [41]:

14 $${C}_{\mathrm{bat}}=\frac{{E}_{\mathrm{l}}\times \mathrm{AD}}{\mathrm{DOD}\times {\eta }_{\mathrm{inv}}\times {\eta }_{\mathrm{b}}},$$

where ${\mathrm{E}}_{\mathrm{l}}$ denotes the total energy load that should be transferred to the HRES; $\mathrm{AD}$ denotes the battery autonomy; $\mathrm{DOD}$ denotes the depth of discharge (percent) that should avoid reducing the battery's storage to its minimum state; and ${\eta }_{\mathrm{inv}}$ and ${\eta }_{\mathrm{inv}}$ denote the inverter and battery efficiency (percent), respectively, considering energy transfer losses.

Without exhibiting any other characteristics, a battery can be assumed to be charged and discharged at the required rate within limits imposed by its state of charge (SOC) and depth of discharge (DOD). At the most basic level, assuming no energy is lost during charge storage and retrieval, the SOC is proportional to the battery current by

15 $$\mathrm{SOC}\left(t\right)=\mathrm{SOC}\left(0\right)+\frac{1}{C}{\int }_{0}^{t}I\left({V}_{\mathrm{bat}},{t}^{^{\prime}}\right)d{t}^{^{\prime}},$$

where $C$ denotes the battery's capacity, $I$ denotes the battery's current, and ${V}_{\mathrm{bat}}$ denotes the battery's voltage.

Batteries in this study are utilized to store energy when energy generation is higher than utilization, and the energy is used when the generation is lower than required. The battery is used because of its effective energy storage capability and higher reliability. A 4.2-MWh lithium-ion battery model is connected to our system, which has an initial state of charge at 100%, and the lowest charge state is 40%. The economic parameters and specifications of the battery are presented in Table 3.

**Table 3:** Battery financial and performance parameters [32–39]

Parameter	Value	Unit
Nominal voltage	600	V
Nominal capacity	4.22	MWh
Roundtrip efficiency	90	%
Maximum capacity	7.03 × 103	Ah
Capital cost	500,000	$
Replacement cost	500,000	$
O&M cost	5000	$
Throughput	21,081,851	kWh
Lifetime	15	Years

A bidirectional converter is also engaged with the system to convert the DC power generated from the battery to AC for load compensation. In contrast, an AC-to-DC formation is utilized to charge the batteries from the other resources connected with the system through a common bus. Five hundred kW converters are used in the system to perform the conversion process with a lifetime of approximately 15 years and approximately 95% efficiency. The parameters expressing the characteristics of the converter are presented in Table 4.

**Table 4:** Converter specifications [32–39]

Parameter	Value	Unit
Capacity	100	kW
Efficiency	95	%
Capital cost	10,000	$
Replacement cost	10,000	$
O&M cost	0	$
Lifetime	15	Years

### Hydrogen generation

Water electrolysis is the most practical process for industrial-scale hydrogen production from renewable resources [42–45]. The alkaline electrolyzer and proton-exchange membrane (PEM) electrolyzer are commercially available, and these low-temperature systems currently dominate the market [45, 46]. Typically, the electrolyzer cell's instantaneous energy efficiency can be calculated as follows:

16 $${\eta }_{\mathrm{c}}=\frac{N{V}_{\mathrm{tn}}I}{{P}_{\mathrm{in}}},$$

where $N$ is the number of cells in the stack, ${V}_{\mathrm{tn}}$ is the thermo-neutral voltage, ${P}_{\mathrm{in}}$ is the total input power to the stack and $I$ is the input current through the cell.

Generally, physically simple types with solid electrolytes such as, polymer electrolyte membrane fuel cell (PEMFC) and solid oxide fuel cell (SOFC) are preferred for smaller-scale energy systems, especially remote areas or islands.

Typically, the hydrogen flow generated by fuel cells is given by the following equation:

17 $${q}_{{H}_{2}}^{\mathrm{req}}=\frac{{N}_{0}{N}_{\mathrm{S}}I}{2FU},$$

where ${q}_{{H}_{2}}^{\mathrm{req}}$ denotes the required amount of hydrogen flow to meet the load. ${N}_{0}$ denotes the number of stacks of fuel cells, ${N}_{\mathrm{S}}$ denotes the number of series cells per stack, and $U$ denotes the use rate. At the most fundamental level, hydrogen storage can be compared to an ideal battery, accepting hydrogen ("charging") and releasing it ("discharging") as needed, within the constraints imposed by its capacity, without regard for pressure, temperature, or dynamics.

At a more sophisticated level, the amount of hydrogen in storage can be expressed as the state of hydrogen (SOH), analogous to the SOC (Eq. 15):

18 $$\mathrm{SOH}\left(t\right)=\mathrm{SOH}\left(0\right)+\frac{1}{{C}_{\mathrm{H}}}{\int }_{0}^{t}\dot{{m}_{\mathrm{H}}}\left(p,{t}^{^{\prime}}\right)d{t}^{^{\prime}},$$

where $\dot{{m}_{\mathrm{H}}}$ is the hydrogen mass flow rate and ${C}_{\mathrm{H}}$ is the gravimetric capacity of the storage and $p$ is the hydrogen pressure.

**Fig. 5:**
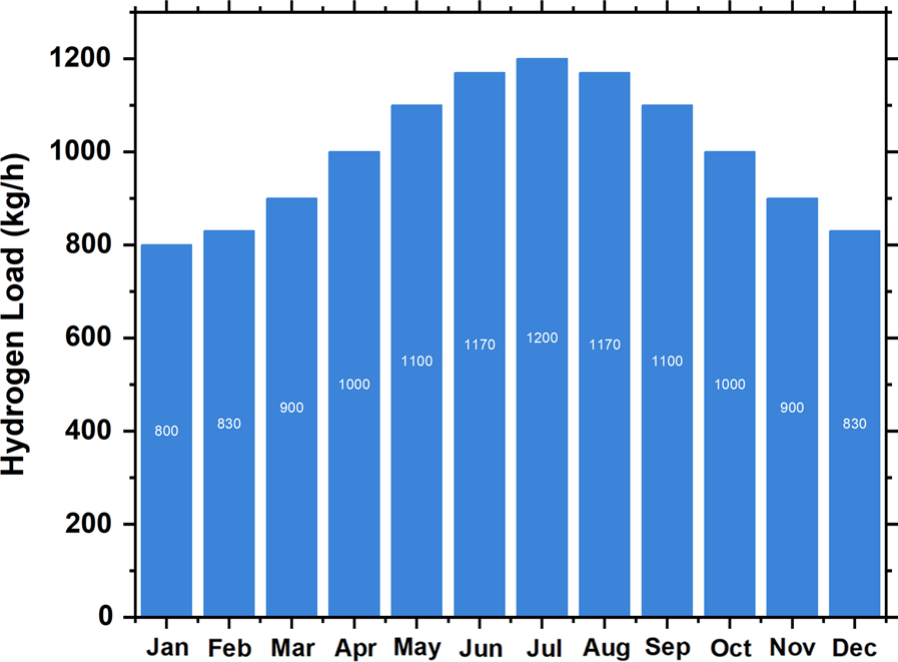
Hydrogen load profile

We scaled the annual daily average to 800 ton/day, and we synthesized the data by considering randomness with daily and hourly noise inputs as shown in Fig. 5. A hydrogen tank's capital and replacement costs are estimated to be 1500 $/kg of hydrogen, with a life expectancy of 25 years (Table 5) [32, 33, 47].

**Table 5:** Economic data for hydrogen system component [32, 33, 47]

Components	Capital cost ($)	Replacement cost ($)	O&M cost ($)	Life time
Fuel cell (FC)	3000	3000	0.030	40,000 h
Electrolyzer	2000	2000	0	15 years
Hydrogen tank	1500	1500	0	25 years

## Results

This study focuses on finding an optimal hybrid power generating system at the location of Yanbu, KSA. KSA has a hot, dry climate and abundant RE resources, particularly solar and wind. The climatic conditions in KSA make it one of the most suitable candidates for utilizing RE resources to compensate for future energy demands while decreasing dependence on oil for power production [48].

Yanbu is an industrial city in the western region of KSA, situated in the Red Sea coastal area, and it has good levels of solar irradiation and great wind potential [49]. The meteorological data of the selected site are presented in Table 6.

**Table 6:** Meteorological data for the selected site

Sr. no.	Particulars	Descriptions
1	Project site	Yanbu, KSA
2	Province	Al Madinah
3	Geographical coordinates	22.7907° N, 39.0190° E
4	Daily global solar irradiance	5.85 kWh/m^2^
5	Annual global solar irradiance	2,135 kWh/m^2^
6	Wind speed	4.72 m/s

Different system structures can be analyzed for energy compensation and performance in delivering the required amount of electrical energy. The economic aspects can be compared to find the optimal solution. Emission generation in the systems is also compared to find the system with the least emissions while having appropriate electrical and economic values.

### Electrical comparison

**Fig. 6:**
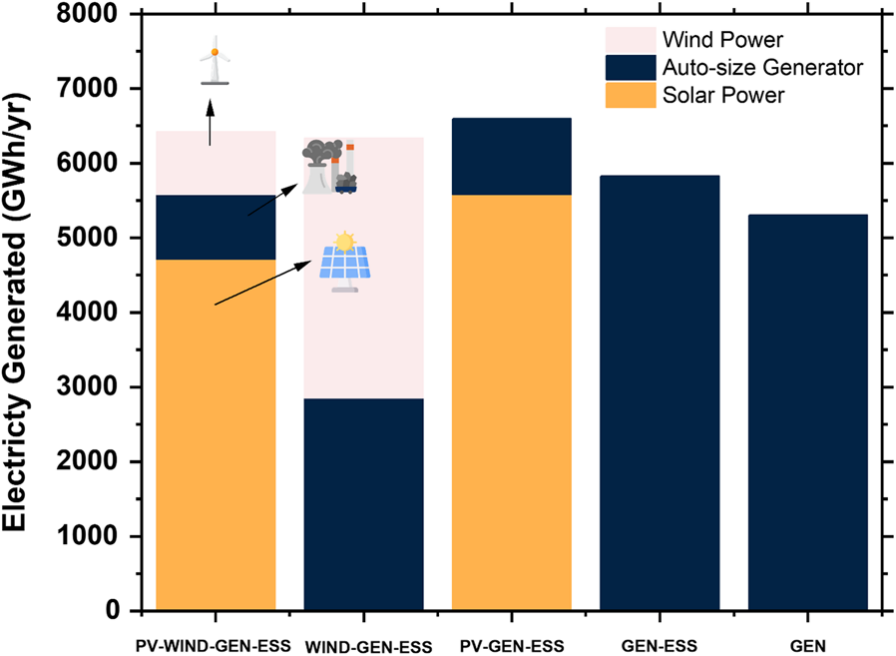
Electrical production distributions and their renewable fractions

The total energy generation and RF in different system configurations are highlighted in Table 7 and presented in Fig. 6. The system with the highest energy generation is PV–Gen–ESS, producing 6,597,135 MWh/year mainly obtained from solar PV, with 5,576,657 MWh/year equivalent to 8.5%. The lowest power generation is provided by generator only.

**Table 7:** Electrical production and renewable fraction summary

System architecture	Solar PV (MWh/year)	Autosize generator (MWh/year)	Wind power (MWh/year)	Total generation (MWh/year)
PV–Wind–Gen–ESS	4,713,856	862,762	842,003	6,418,622
Wind–Gen–ESS	–	2,855,311	3,479,561	6,334,872
PV–Gen–ESS	5,576,657	1,020,473	–	6,597,135
Gen–ESS	–	5,825,214	–	5,825,214
Gen only	–	5,300,219	–	5,300,219

The energy that is not consumed by the load and stored by the storage devices is available as excess energy. Excess energy assessment in different system configurations is summarized in Table 8. It is evident from the excess energy summary that the highest amount of excess energy generation is in the PV–Gen ESS system configuration, whereas the least excess energy generation is in the Gen-only configuration.

**Table 8:** Excess energy summary

System architecture	Excess electricity generation (MWh/year)	Percent (%)
PV–Wind–Gen–ESS	599,357	9.34
Wind–Gen–ESS	648,111	10.2
PV–Gen–ESS	702,099	10.6
Gen–ESS	0	0
Gen only	7718	0.15

### Economic comparison

The economic comparison of the different system configurations reflects that the highest COE of 0.190 is attained with the Gen-only system, while the lowest COE is seen in the PV–Wind–Gen–ESS system at $0.10/kWh. The operating cost and NPC are also minimal in the configuration PV–Wind–Gen–ESS. An economic comparison of the different system configurations is presented in Table 9.

**Table 9:** Economic comparison of different system configurations

System architecture	Capital cost (billion $)	NPC (billion $)	COE/kWh (S)	Operating cost (million $)
PV–Wind–Gen–ESS	4.58	6.80	0.0993	151
Wind–Gen–ESS	3.13	7.63	0.112	348
PV–Gen–ESS	4.85	6.96	0.102	163
Gen–ESS	1.15	9.38	0.137	637
Gen only	0.650	13.0	0.190	957

### Fuel consumption and renewable fraction comparison

Fuel consumption refers to the amount of fuel consumed by each system configuration for generating the required amount of energy. The fuel price used in this study is $0.14/L [50]. The fuel consumption and RFs in different system configurations are presented in Table 10. It is evident from the results that the highest fuel consumption is in the Gen–ESS system, whereas the least fuel consumption is in the PV–Wind–Gen–ESS system.

**Table 10:** Fuel consumption and renewable fraction comparison

System architecture	Avg. fuel per hour (L/hour)	Avg. fuel per day (L/day)	Total fuel consumed (L)	Renewable fraction (%)
PV–Wind–Gen–ESS	24,721	593,310	216,557,988	83.7
Wind–Gen–ESS	81,836	1,964,056	716,880,587	46
PV–Gen–ESS	29,209	701,021	255,872,836	80.7
Gen–ESS	166,878	4,005,072	1,461,851,188	0
Gen only	161,846	3,884,303	1,417,770,618	0

The RF depends on the energy generation from RE resources, which is highest in the case of PV–Wind–Gen–ESS and is 83.7% of the total energy generation. The least RF is common for both the Gen–ESS and Gen-only systems as there are no RE resources in these system configurations.

### Emissions generated

The power sector is critical in any scenario involving significant economic decarbonization. The sector's emissions must be significantly reduced even as demand for electricity increases as other sectors transition from fossil fuels to electricity to reduce their carbon emissions. Since 2008, the power sector has been decarbonizing at an average annual rate of 3% due to increased generation from natural gas, wind, and solar. Under a business-as-usual scenario, electricity generation is projected to increase by 24% by 2050. Decarbonizing the power sector requires a multifaceted approach that may include a continued substitution of zero- or low-emission energy sources, continued improvements in end-use efficiency, increased grid flexibility and storage, and continued use of carbon capture, utilization, and storage (CCUS) in the remaining fossil fuel-based generation [51]. Carbon dioxide (CO_2_) emissions from the U.S. electric power sector were 1447 million metric tons (MMmt) in 2020, accounting for approximately 32% of total U.S. energy-related CO_2_ emissions of 4575 MMmt [52]. This leaves significant opportunities for electricity decarbonization.

**Fig. 7:**
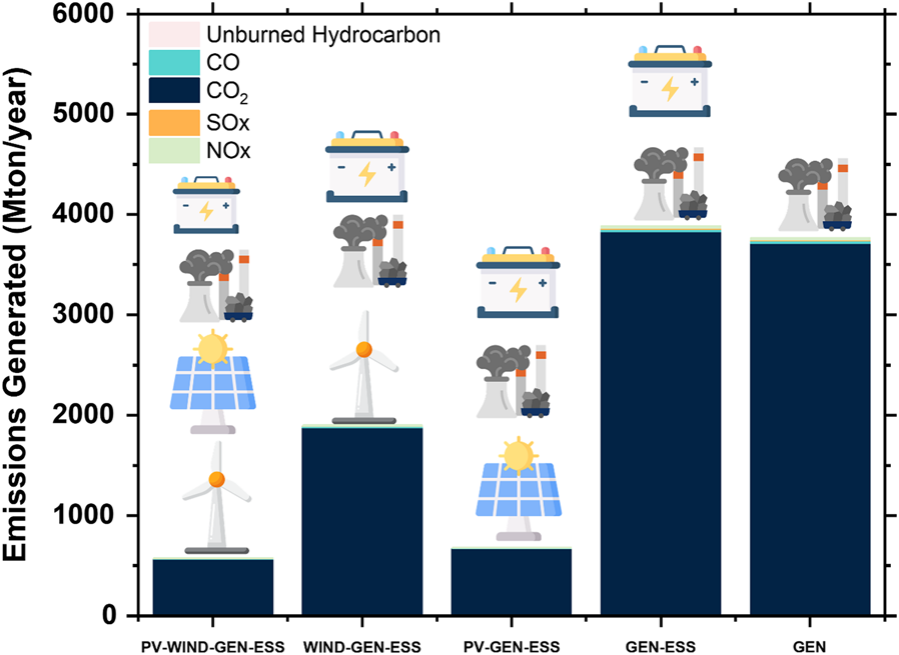
Emissions generated from each microgrid design scenario

Comparison of emissions among the system configurations is carried out to select the system with the least GHG emissions, thus having the least environmental impact. The PV–Wind–Gen–ESS system has the least GHG emissions, whereas the emissions are highest in the Gen–ESS system configuration (Fig. 7). A comparison of emissions among the configurations is presented in Table 11.

**Table 11:** Emissions comparison among system configurations

System architecture	CO_2_ (ton/year)	CO (ton/year)	Unburned hydrocarbons (ton/year)	Particulate matter (ton/year)	SOx (ton/year)	NOx (ton/year)
PV–Wind–Gen–ESS	566,865	3573	156	22	1388	3357
Wind–Gen–ESS	1,876,517	11,829	516	72	4595	11,112
PV–Gen–ESS	669,776	4221	184	26	1640	3966
Gen–ESS	3,826,563	24,121	1053	146	9370	22,659
Gen only	3,711,177	23,393	1021	142	9088	21,975

## Discussion

### Optimal system configuration

The optimal microgrid design identified in this study is the scenario of PV/wind/battery/generator with an NPC of $6.8 billion and an LCOE of $0.1/kWh for energy costs. The optimal microgrid system for this project is 514,127 m^2^ of PV panels achieved by installing 264,966 solar panels coupled with an 862,762 MWh/year autosize generator.

**Fig. 8:**
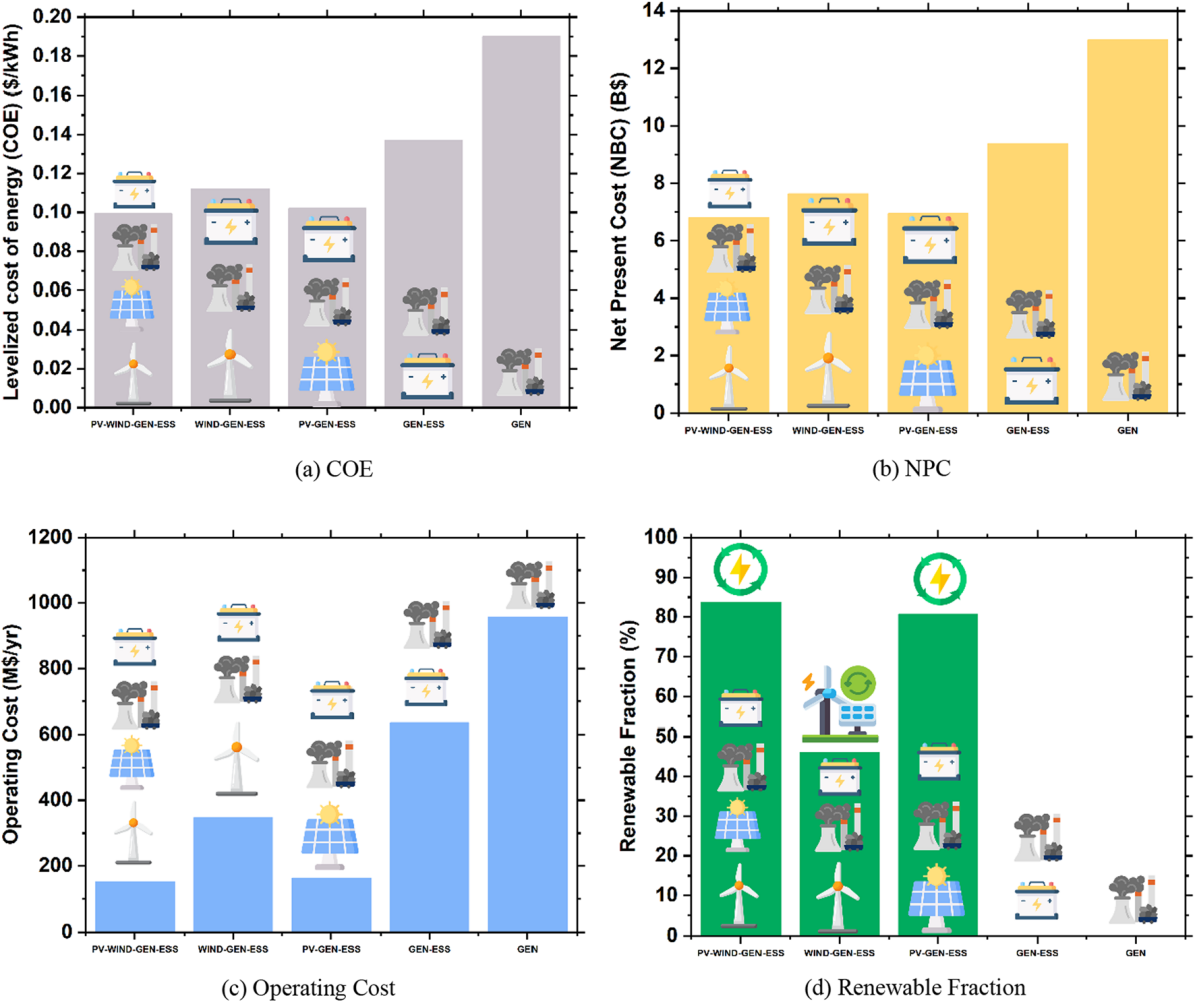
Summary of system parameter comparisons. **a** COE, **b** NPC, **c** operating cost, **d** renewable fraction

These electrical, economic, and emissions comparison results demonstrate that the PV–Wind–Gen–ESS system is the optimal system configuration with the constraint of least GHG emissions. Financial elements such as COE, NPC, and operating cost are the least in this configuration, and emissions are reduced by a significant margin. Emissions are reduced as the RF increases, and the generator's power generation is decreased with less fuel consumption. A comparison of these financial resources and renewable fractions between all the system configurations discussed in this paper is presented in Fig. 8.

### Hydrogen production

The adoption of hydrogen as a clean energy source is still in its infancy and faces significant economic constraints in its development. Green hydrogen production is relatively expensive, but its production in the near future shows a bright picture due to the recent drop in costs of renewable energy.

**Fig. 9:**
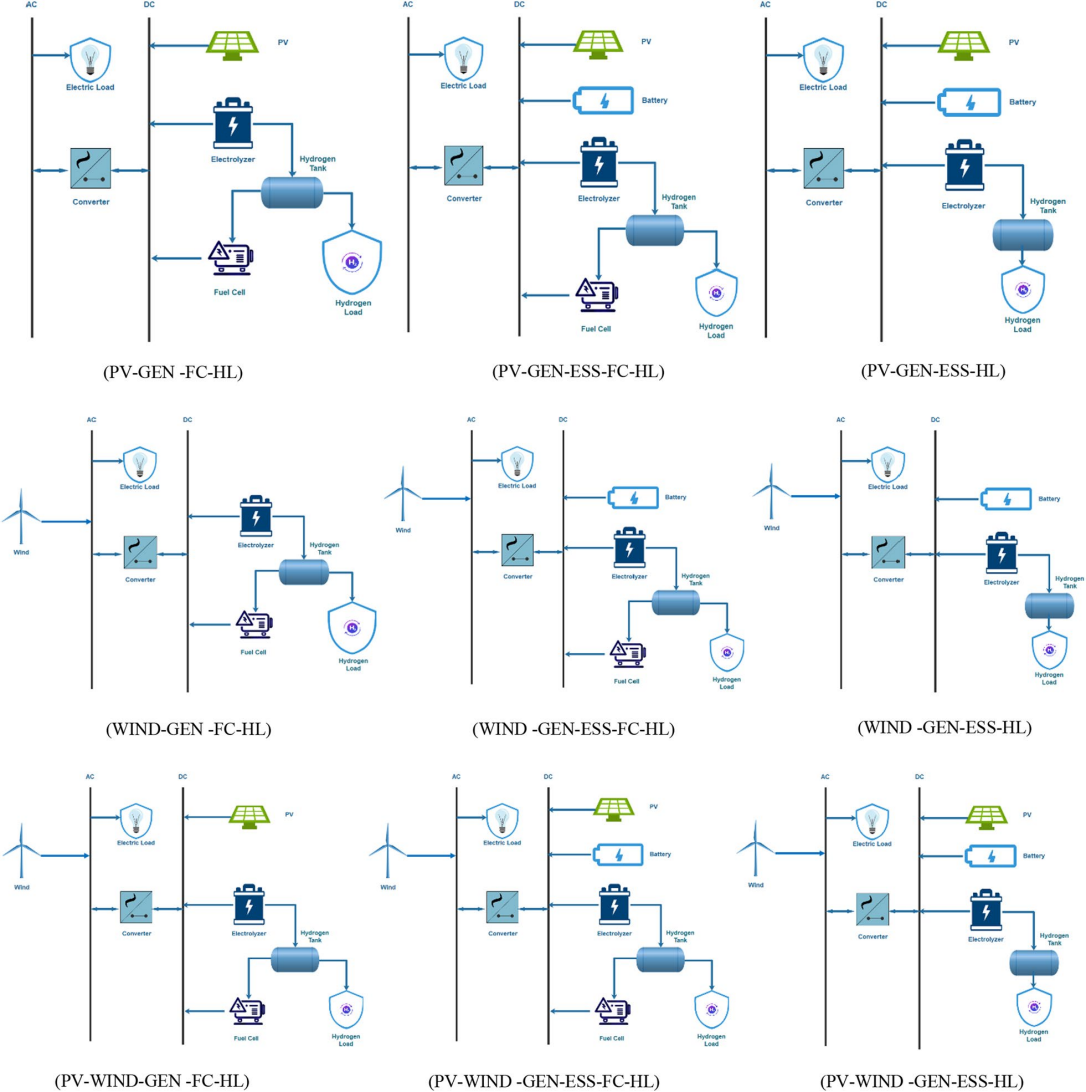
Schematic representation of different system configurations for hydrogen production without a battery bank, with a battery bank and without a fuel cell

In addition to the previous analysis, we investigate three distinct scenarios for each energy system (photovoltaic, wind or a mix of the two) from Fig. 2a, b, and c, including energy systems without battery bank storage but with a fuel cell; battery bank storage with a fuel cell; and battery bank storage without a fuel cell to determine which combination resulted in the lowest COE for meeting 100% load without loss at the same location (Fig. 9). While hydrogen is the preferred long-term energy storage option (high capacity but low delivery rate relative to capacity), short-term storage of electricity (low capacity but high delivery rate relative to capacity) is required to accommodate brief surges in demand and to extend the life of the fuel cell by smoothing the demand for hydrogen.

The results (Tables 12 and 13) indicate that, for each energy system (photovoltaic, wind, or a mix of the two), LCOE and NPC are minimal in the systems with no fuel cell but with a battery equivalent to $0.145/kWh (PV–WIND–GEN–ESS–HL) higher than the optimum system selected in the previous section that store energy in batteries instead of hydrogen at $0.1/kWh. Operating the energy system without batteries and with only fuel cells and hydrogen electrolyzers increases LCOE from 0.145 to $0.24/kWh. Adding fuel cell components to the PV–WIND–GEN–ESS–HL configuration reduces the LCOH 29.6 from 44.9 to $25.6/kg H_2_ (PV–WIND–GEN–ESS–FC–HL). Among the systems, there is a tradeoff between LCOE and LCOH, PV–WIND–GEN–ESS–HL produced the lowest LCOE, and PV–WIND–GEN–ESS–FC–HL produced the lowest LCOH. For the purpose of this project, PV–WIND–GEN–ESS–FC–HL is selected as the optimum condition as it produces the lowest LCOH.

**Table 12:** Economic comparison of different system configurations with hydrogen production

System architecture	Capital cost (B$)	NPC (B$)	LCOE ($/kWh)	Operating cost (M$/year)
PV–GEN–FC–HL	4.40	17.48	0.258	1022
PV–GEN–ESS–HL	8.77	11.1	0.162	181
PV–GEN–ESS–FC–HL	9.25	11.5	0.168	173
PV–WIND–GEN–ESS–HL	6.21	9.93	0.145	288
PV–WIND–GEN–FC–HL	5.43	16.6	0.242	863
PV–WIND–GEN–ESS–FC–HL	7.31	10.6	0.155	256
GEN–WIND–FC–HL	4.43	16.6	0.243	863
GEN–ESS–WIND–HL	7.32	11.8	0.172	346
GEN–ESS–WIND–FC–HL	9.28	13.2	0.193	303

**Table 13:** Hydrogen production summary for different system configuration

System architecture	Hydrogen production (tons/year)	LCOH ($)
PV–GEN–FC–HL	26,122	52.2
PV–GEN–ESS–HL	26,044	33.0
PV–GEN–ESS–FC–HL	26,510	33.5
PV–WIND–FC–HL	28,562	44.9
PV–WIND–GEN–ESS–HL	25,989	29.6
PV–WIND–GEN–ESS–FC–HL	32,132	25.6
GEN–WIND–FC–HL	28,562	44.9
GEN–ESS–WIND–HL	25,968	35.1
GEN–ESS–WIND–FC–HL	32,887	31.0

Integrating green hydrogen production electrolyzers with dedicated solar or wind power plants (RES) can result in significant cost reductions, making green hydrogen pathways a viable option in the years to come. With solar PV and wind generation costs declining, building electrolyzers in locations with excellent renewable resource conditions, such as KSA, could become a low-cost hydrogen supply option, even when accounting for the transmission and distribution costs associated with transporting hydrogen from (often remote) renewable resource locations to end-users. Using our analysis, the optimum configurations can generate up to 32,132 tons of hydrogen per year (tH_2_/year), and 380,824 tons per year of CO_2_ emissions can be avoided.

The Kingdom is perfectly positioned to capitalize on hydrogen production because of its renewable photovoltaic generation. Moreover, it presents a strong case for green hydrogen adoption, given that the country has abundantly available land and a high capacity factor for the deployment of renewable energy. This renewable energy can be used for clean hydrogen generation, supplying consumers with clean or low-carbon electricity. The decrease in renewable energy costs may also impact the costs of green hydrogen. In addition, hydrogen gas can be utilized as a fuel in transport vehicles or as a feedstock for industrial processes. Ammonia can also be generated from this clean hydrogen which Saudi Arabia can then export with global green hydrogen demand. The cost of green hydrogen and blue hydrogen are influenced by the costs and efficiency of electrolysis and carbon capture technology, respectively. These factors will provide profitability from clean hydrogen production to Saudi Arabia [53].

## Conclusion

The hybrid microgrid isolated system is a cost-effective solution, particularly in KSA, which receives significant solar radiation. This article discusses the design and implementation of three hybrid microgrid systems in the Yanbu region. The NPC for this project is $10.6 billion, and the LCOE is $0.155/kWh while LCOH is $25.6/kg H_2_. Different system configurations are analyzed in the study to find an optimal system with the least GHG emissions and least costly parameters. The PV–WIND–GEN–ESS–FC–HL system configuration is selected as the optimal system configuration with better environmental and economic benefits than any of the others considered in this paper. This study reveals that optimum configuration can generate up to 32,132 tons of hydrogen per year (tH_2_/year), and 380,824 tons per year of CO_2_ emissions can be avoided. By 2030, 10% of installed capacity (250 GW) is expected to be converted to hydrogen fuel, resulting in an annual increase in hydrogen demand of 120–170 million tons. Compared to today's hydrogen consumption of 70 million tons per year, the enormous potential of the future hydrogen market is apparent.

Future work's primary focus will be developing and implementing novel optimization algorithms and microgrid system configurations that incorporate efficient algorithms and effective power management aids in creating a cost-effective microgrid system with hydrogen production and CO_2_ capturing facilities.

## Data Availability

Not applicable.

## List of symbols

*A*_g_: Coefficient of consumption curve (a = 0.246 L/kW)
*A*_pv_: Area covered by the PV panels (m^2^)
*A*_pv_: The area of the photovoltaic panel
*A*_tt_: Cross-sectional area of the tidal (m^2^)
*A*_wind_: Swept area by the wind turbine (m^2^)
*C*_p_: Maximum power coefficient (%)
*P*_pv_: Output power of the PV (kW)
*P*_r_: Rated power (kW)
*P*_wind_: Output power of the wind turbine (kW)
*T*: Temperature °C
*T*_a_: Ambient temperature (°C)
*T*_r_: Reference temperature of solar cell °C
*V*: Wind speed (m/s)
*V*_ci_: Cut-in wind speed (m/s)
*V*_co_: Cut-out wind speed (m/s)
*V*_r_: Rated wind speed (m/s)
$${\eta }_{\rm{b}}$$: Efficiency of the battery (%)
$${\eta }_{\rm{inv}}$$: Efficiency of the inverter (%)
$${\eta }_{\rm{pv}}$$: Efficiency of the PV system (%)
$${\eta }_{\rm{r}}$$: Reference efficiency of PV panels (%)
$${\eta }_{\rm{t}}$$: Efficiency MPPT system (%)
C: Capital cost ($)
NOCT: Nominal operating cell temperature (°C)
NPC: Net present cost ($)
OM: Maintenance and operation ($)
$$A$$: Availability index
$$I$$: Solar irradiation (kW/m^2^)
$$\beta$$: Temperature coefficient (0.004 to 0.006 °C)
$$\rho$$: Air density (kg/m^3^)

## Abbreviations

ACS: Annualized cost of the system
BESS: Battery energy storage system
COE: Cost of energy
HMGs: Hybrid microgrid system
HOMER: Hybrid optimization of multiple energy resources
HRES: Hybrid renewable energy systems
LCOE: Levelized cost of energy
LCOH: Levelized cost of hydrogen [$]
NPC: Net present cost
PV: Photovoltaic
RF: Renewable fraction
